# 1-[(2-Anilinoeth­yl)iminiometh­yl]-2-naph­thol­ate

**DOI:** 10.1107/S1600536809019096

**Published:** 2009-05-23

**Authors:** Yu-Mei Hao

**Affiliations:** a Department of Chemistry, Baicheng Normal University, Baicheng 137000, People’s Republic of China

## Abstract

The title Schiff base compound, C_19_H_18_N_2_O, was prepared by the reaction of equimolar quanti­ties of 2-hydr­oxy-1-naphthaldehyde with *N*-phenyl­ethane-1,2-diamine in a methanol solution. The mol­ecule adopts a zwitterionic conformation with the naphthyl OH group deprotonated and the imine N atom protonated. An intra­molecular N—H⋯O hydrogen bond forms between them. The dihedral angle between the benzene ring and the naphthyl system is 86.9 (2)°. In the crystal structure, mol­ecules are linked through inter­molecular N—H⋯O hydrogen bonds, forming chains running along the *b* axis.

## Related literature

For the pharmaceutical and medicinal activity of Schiff bases, see: Dao *et al.* (2000 ▶); Sriram *et al.* (2006 ▶); Karthikeyan *et al.* (2006 ▶). For Schiff base coordination chemistry, see: Ali *et al.* (2008 ▶); Kargar *et al.* (2009 ▶); Yeap *et al.* (2009 ▶). For related structures, see: Fun *et al.* (2009 ▶); Nadeem *et al.* (2009 ▶); Eltayeb *et al.* (2008 ▶). For reference structural data, see: Allen *et al.* (1987 ▶).
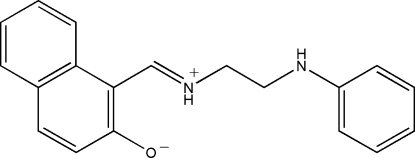


## Experimental

### 

#### Crystal data

C_19_H_18_N_2_O
*M* *_r_* = 290.35
Monoclinic, 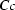

*a* = 27.511 (3) Å
*b* = 6.845 (2) Å
*c* = 8.543 (2) Å
β = 104.263 (2)°
*V* = 1559.2 (6) Å^3^
*Z* = 4
Mo *K*α radiation
μ = 0.08 mm^−1^
*T* = 298 K
0.23 × 0.21 × 0.18 mm

#### Data collection

Bruker SMART CCD area-detector diffractometer
Absorption correction: multi-scan (*SADABS*; Sheldrick, 1996 ▶) *T*_min_ = 0.982, *T*_max_ = 0.986
4485 measured reflections
1753 independent reflections
1312 reflections with *I* > 2σ(*I*)
*R*_int_ = 0.021

#### Refinement

*R*[*F* ^2^ > 2σ(*F* ^2^)] = 0.038
*wR*(*F* ^2^) = 0.105
*S* = 1.06
1753 reflections
202 parameters
3 restraints
H atoms treated by a mixture of independent and constrained refinement
Δρ_max_ = 0.18 e Å^−3^
Δρ_min_ = −0.18 e Å^−3^

### 

Data collection: *SMART* (Bruker, 2002 ▶); cell refinement: *SAINT* (Bruker, 2002 ▶); data reduction: *SAINT*; program(s) used to solve structure: *SHELXS97* (Sheldrick, 2008 ▶); program(s) used to refine structure: *SHELXL97* (Sheldrick, 2008 ▶); molecular graphics: *SHELXTL* (Sheldrick, 2008 ▶); software used to prepare material for publication: *SHELXL97*.

## Supplementary Material

Crystal structure: contains datablocks global, I. DOI: 10.1107/S1600536809019096/sj2625sup1.cif

Structure factors: contains datablocks I. DOI: 10.1107/S1600536809019096/sj2625Isup2.hkl

Additional supplementary materials:  crystallographic information; 3D view; checkCIF report

## Figures and Tables

**Table 1:** Hydrogen-bond geometry (Å, °)

*D*—H⋯*A*	*D*—H	H⋯*A*	*D*⋯*A*	*D*—H⋯*A*
N1—H1⋯O1	0.907 (10)	1.84 (3)	2.582 (3)	137 (3)
N2—H2⋯O1^i^	0.86	2.43	3.043 (3)	129

Symmetry code: (i) 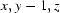.

**Fig. 1.**
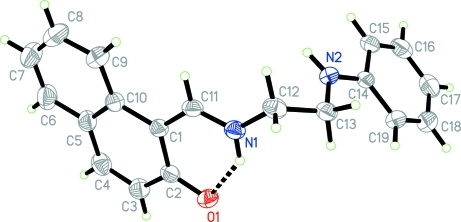
The molecular structure of (I) with 30% probability ellipsoids. The intramolecular N–H···O hydrogen bond is shown as a dashed line.

**Fig. 2.**
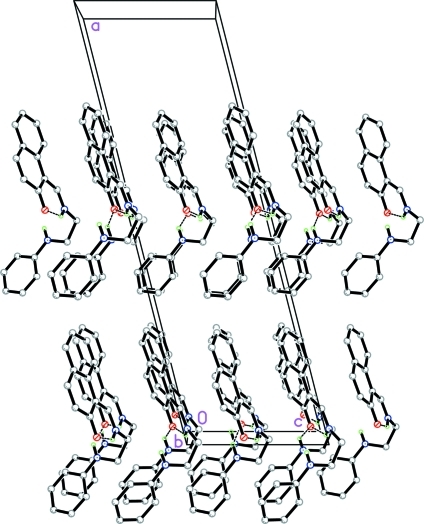
Molecular packing of (I) with hydrogen bonds drawn as dashed lines.

## supplementary crystallographic information

### Comment

Schiff base compounds are an important class of materials used in the pharmaceutical and medicinal fields (Dao *et al.*, 2000; Sriram *et al.*, 2006; Karthikeyan *et al.*, 2006). They are also used as versatile ligands in coordination chemistry (Ali *et al.*, 2008; Kargar *et al.*, 2009; Yeap *et al.*, 2009). Recently, the crystal structures of several Schiff base compounds have been reported (Fun *et al.*, 2009; Nadeem *et al.*, 2009; Eltayeb *et al.*, 2008). In this paper, the new Schiff base title compound, (I), Fig. 1, is reported.

In (I), the H atom of the phenol group is transferred to the imine N atom, forming an intramolecular N–H···O hydrogen bond (Table 1). The dihedral angle between the benzene ring and the naphthyl ring is 86.9 (2)°. All the bond lengths are within normal values (Allen *et al.*, 1987). In the crystal structure of the compound, molecules are linked through intermolecular N–H···O hydrogen bonds (Table 1), forming chains running along the b axis (Fig. 2).

### Experimental

2-Hydroxy-1-naphthylaldehyde (0.1 mmol, 17.2 mg) and N-phenylethane-1,2-diamine (0.1 mmol, 13.6 mg) were refluxed in a 30 ml methanol solution for 30 min to give a clear orange solution. Yellow block-shaped single crystals of the compound were formed by slow evaporation of the solvent over several days at room temperature.

### Refinement

In the absence of significant anomalous dispersion effects, 1421 Freidel pairs were merged. H1 was located from a difference Fourier map and refined isotropically, with the N–H distance restrained to 0.90 (1)Å, and with *U*_iso_ restrained to 0.08Å^2^. Other H atoms were constrained to ideal geometries, with d(C–H) = 0.93–0.97Å, d(N–H) = 0.86Å, and with *U*_iso_(H) = 1.2*U*_eq_(C,N).

### Crystal data

**Table:**

C_19_H_18_N_2_O	*F*(000) = 616
*M**_r_* = 290.35	*D*_x_ = 1.237 Mg m^−^^3^
Monoclinic, *C**c*	Mo *K*α radiation, λ = 0.71073 Å
Hall symbol: C -2yc	Cell parameters from 1258 reflections
*a* = 27.511 (3) Å	θ = 2.5–24.5°
*b* = 6.845 (2) Å	µ = 0.08 mm^−^^1^
*c* = 8.543 (2) Å	*T* = 298 K
β = 104.263 (2)°	Block, yellow
*V* = 1559.2 (6) Å^3^	0.23 × 0.21 × 0.18 mm
*Z* = 4	

### Data collection

**Table:**

Bruker SMART CCD area-detector diffractometer	1753 independent reflections
Radiation source: fine-focus sealed tube	1312 reflections with *I* > 2σ(*I*)
graphite	*R*_int_ = 0.021
ω scans	θ_max_ = 27.5°, θ_min_ = 3.1°
Absorption correction: multi-scan (*SADABS*; Sheldrick, 1996)	*h* = −31→35
*T*_min_ = 0.982, *T*_max_ = 0.986	*k* = −6→8
4485 measured reflections	*l* = −11→11

### Refinement

**Table:**

Refinement on *F*^2^	Primary atom site location: structure-invariant direct methods
Least-squares matrix: full	Secondary atom site location: difference Fourier map
*R*[*F*^2^ > 2σ(*F*^2^)] = 0.038	Hydrogen site location: inferred from neighbouring sites
*wR*(*F*^2^) = 0.105	H atoms treated by a mixture of independent and constrained refinement
*S* = 1.06	*w* = 1/[σ^2^(*F*_o_^2^) + (0.0531*P*)^2^ + 0.1092*P*] where *P* = (*F*_o_^2^ + 2*F*_c_^2^)/3
1753 reflections	(Δ/σ)_max_ < 0.001
202 parameters	Δρ_max_ = 0.18 e Å^−^^3^
3 restraints	Δρ_min_ = −0.18 e Å^−^^3^

### Special details

**Table:**

Geometry. All esds (except the esd in the dihedral angle between two l.s. planes) are estimated using the full covariance matrix. The cell esds are taken into account individually in the estimation of esds in distances, angles and torsion angles; correlations between esds in cell parameters are only used when they are defined by crystal symmetry. An approximate (isotropic) treatment of cell esds is used for estimating esds involving l.s. planes.
Refinement. Refinement of F^2^ against ALL reflections. The weighted R-factor wR and goodness of fit S are based on F^2^, conventional R-factors R are based on F, with F set to zero for negative F^2^. The threshold expression of F^2^ > 2sigma(F^2^) is used only for calculating R-factors(gt) etc. and is not relevant to the choice of reflections for refinement. R-factors based on F^2^ are statistically about twice as large as those based on F, and R- factors based on ALL data will be even larger.

### Fractional atomic coordinates and isotropic or equivalent isotropic displacement parameters (Å^2^)

**Table:**

	*x*	*y*	*z*	*U*_iso_*/*U*_eq_	
O1	0.52924 (7)	0.9086 (3)	0.4162 (2)	0.0576 (5)	
N1	0.53391 (8)	0.5581 (3)	0.5306 (3)	0.0497 (5)	
N2	0.46277 (8)	0.2671 (3)	0.3271 (3)	0.0522 (6)	
H2	0.4912	0.2202	0.3207	0.063*	
C1	0.60256 (9)	0.7135 (4)	0.4543 (3)	0.0465 (6)	
C2	0.57425 (10)	0.8886 (4)	0.4038 (3)	0.0491 (6)	
C3	0.59731 (11)	1.0404 (4)	0.3330 (4)	0.0608 (7)	
H3	0.5793	1.1537	0.2974	0.073*	
C4	0.64475 (12)	1.0231 (5)	0.3167 (4)	0.0658 (8)	
H4	0.6583	1.1247	0.2689	0.079*	
C5	0.67481 (11)	0.8544 (5)	0.3702 (3)	0.0583 (7)	
C6	0.72446 (13)	0.8429 (6)	0.3537 (5)	0.0791 (10)	
H6	0.7374	0.9433	0.3027	0.095*	
C7	0.75394 (13)	0.6840 (7)	0.4128 (5)	0.0908 (12)	
H7	0.7868	0.6775	0.4031	0.109*	
C8	0.73446 (14)	0.5358 (7)	0.4859 (6)	0.0953 (13)	
H8	0.7546	0.4294	0.5269	0.114*	
C9	0.68608 (12)	0.5403 (5)	0.5000 (5)	0.0748 (9)	
H9	0.6738	0.4360	0.5487	0.090*	
C10	0.65441 (10)	0.6995 (4)	0.4422 (3)	0.0541 (7)	
C11	0.57962 (10)	0.5575 (4)	0.5134 (3)	0.0490 (6)	
H11	0.5985	0.4445	0.5427	0.059*	
C12	0.50873 (11)	0.3947 (4)	0.5875 (4)	0.0568 (7)	
H12A	0.5046	0.4237	0.6945	0.068*	
H12B	0.5293	0.2784	0.5948	0.068*	
C13	0.45822 (10)	0.3570 (4)	0.4749 (4)	0.0540 (7)	
H13A	0.4389	0.2724	0.5278	0.065*	
H13B	0.4403	0.4796	0.4504	0.065*	
C14	0.42170 (10)	0.2552 (4)	0.1944 (3)	0.0477 (6)	
C15	0.42493 (12)	0.1449 (4)	0.0608 (4)	0.0590 (7)	
H15	0.4542	0.0759	0.0627	0.071*	
C16	0.38583 (14)	0.1359 (4)	−0.0735 (4)	0.0689 (9)	
H16	0.3890	0.0605	−0.1609	0.083*	
C17	0.34208 (13)	0.2356 (5)	−0.0821 (5)	0.0732 (9)	
H17	0.3157	0.2291	−0.1742	0.088*	
C18	0.33809 (12)	0.3459 (5)	0.0492 (4)	0.0687 (8)	
H18	0.3088	0.4154	0.0454	0.082*	
C19	0.37702 (11)	0.3545 (4)	0.1862 (4)	0.0579 (7)	
H19	0.3734	0.4276	0.2743	0.070*	
H1	0.5170 (12)	0.671 (3)	0.501 (4)	0.080*	

### Atomic displacement parameters (Å^2^)

**Table:**

	*U*^11^	*U*^22^	*U*^33^	*U*^12^	*U*^13^	*U*^23^
O1	0.0519 (11)	0.0450 (10)	0.0754 (13)	0.0099 (8)	0.0146 (9)	0.0058 (9)
N1	0.0534 (14)	0.0385 (12)	0.0542 (13)	0.0029 (9)	0.0075 (11)	0.0009 (9)
N2	0.0450 (12)	0.0432 (12)	0.0711 (15)	0.0038 (9)	0.0196 (11)	−0.0043 (10)
C1	0.0464 (14)	0.0426 (13)	0.0468 (13)	0.0018 (11)	0.0048 (11)	−0.0035 (11)
C2	0.0530 (16)	0.0412 (14)	0.0482 (15)	0.0029 (11)	0.0032 (12)	−0.0041 (11)
C3	0.0627 (18)	0.0502 (17)	0.0656 (19)	0.0018 (13)	0.0084 (15)	0.0088 (14)
C4	0.068 (2)	0.0579 (18)	0.0687 (19)	−0.0112 (14)	0.0112 (16)	0.0064 (15)
C5	0.0512 (15)	0.064 (2)	0.0569 (16)	−0.0055 (13)	0.0081 (13)	−0.0087 (13)
C6	0.058 (2)	0.092 (3)	0.088 (2)	−0.0136 (18)	0.0210 (18)	−0.009 (2)
C7	0.054 (2)	0.107 (3)	0.112 (3)	0.005 (2)	0.021 (2)	−0.014 (3)
C8	0.063 (2)	0.087 (3)	0.136 (4)	0.025 (2)	0.024 (2)	0.004 (3)
C9	0.0580 (19)	0.067 (2)	0.098 (2)	0.0130 (15)	0.0163 (18)	0.0022 (19)
C10	0.0495 (15)	0.0532 (16)	0.0548 (16)	0.0020 (12)	0.0039 (12)	−0.0087 (12)
C11	0.0533 (16)	0.0392 (14)	0.0500 (14)	0.0088 (11)	0.0041 (11)	−0.0025 (11)
C12	0.0704 (18)	0.0447 (14)	0.0568 (16)	0.0015 (13)	0.0184 (14)	0.0058 (13)
C13	0.0594 (16)	0.0397 (14)	0.0665 (17)	−0.0028 (12)	0.0226 (14)	−0.0025 (12)
C14	0.0474 (15)	0.0345 (12)	0.0659 (17)	−0.0024 (10)	0.0230 (14)	0.0004 (11)
C15	0.0647 (17)	0.0372 (13)	0.081 (2)	−0.0035 (12)	0.0301 (16)	−0.0078 (13)
C16	0.086 (2)	0.0496 (18)	0.074 (2)	−0.0129 (17)	0.0246 (19)	−0.0135 (15)
C17	0.074 (2)	0.0609 (19)	0.079 (2)	−0.0137 (17)	0.0080 (17)	0.0017 (17)
C18	0.0557 (18)	0.0620 (19)	0.087 (2)	0.0049 (15)	0.0152 (17)	0.0037 (17)
C19	0.0548 (16)	0.0503 (17)	0.0725 (19)	0.0072 (12)	0.0228 (14)	−0.0027 (13)

### Geometric parameters (Å, °)

**Table:**

O1—C2	1.276 (3)	C8—C9	1.366 (5)
N1—C11	1.302 (3)	C8—H8	0.9300
N1—C12	1.460 (3)	C9—C10	1.407 (4)
N1—H1	0.907 (10)	C9—H9	0.9300
N2—C14	1.392 (3)	C11—H11	0.9300
N2—C13	1.437 (3)	C12—C13	1.505 (4)
N2—H2	0.8600	C12—H12A	0.9700
C1—C11	1.397 (4)	C12—H12B	0.9700
C1—C2	1.436 (4)	C13—H13A	0.9700
C1—C10	1.459 (4)	C13—H13B	0.9700
C2—C3	1.427 (4)	C14—C15	1.389 (4)
C3—C4	1.351 (4)	C14—C19	1.391 (4)
C3—H3	0.9300	C15—C16	1.367 (5)
C4—C5	1.428 (5)	C15—H15	0.9300
C4—H4	0.9300	C16—C17	1.370 (5)
C5—C6	1.409 (4)	C16—H16	0.9300
C5—C10	1.410 (4)	C17—C18	1.379 (5)
C6—C7	1.376 (6)	C17—H17	0.9300
C6—H6	0.9300	C18—C19	1.379 (4)
C7—C8	1.368 (6)	C18—H18	0.9300
C7—H7	0.9300	C19—H19	0.9300
			
C11—N1—C12	125.9 (2)	C5—C10—C1	118.9 (2)
C11—N1—H1	114 (2)	N1—C11—C1	125.0 (2)
C12—N1—H1	120 (2)	N1—C11—H11	117.5
C14—N2—C13	120.8 (2)	C1—C11—H11	117.5
C14—N2—H2	119.6	N1—C12—C13	111.0 (2)
C13—N2—H2	119.6	N1—C12—H12A	109.4
C11—C1—C2	119.1 (2)	C13—C12—H12A	109.4
C11—C1—C10	120.8 (2)	N1—C12—H12B	109.4
C2—C1—C10	120.1 (2)	C13—C12—H12B	109.4
O1—C2—C3	119.9 (2)	H12A—C12—H12B	108.0
O1—C2—C1	122.0 (2)	N2—C13—C12	111.6 (2)
C3—C2—C1	118.0 (2)	N2—C13—H13A	109.3
C4—C3—C2	121.4 (3)	C12—C13—H13A	109.3
C4—C3—H3	119.3	N2—C13—H13B	109.3
C2—C3—H3	119.3	C12—C13—H13B	109.3
C3—C4—C5	122.4 (3)	H13A—C13—H13B	108.0
C3—C4—H4	118.8	C15—C14—C19	117.3 (3)
C5—C4—H4	118.8	C15—C14—N2	119.9 (2)
C6—C5—C10	120.1 (3)	C19—C14—N2	122.8 (2)
C6—C5—C4	120.8 (3)	C16—C15—C14	121.1 (3)
C10—C5—C4	119.1 (3)	C16—C15—H15	119.4
C7—C6—C5	120.4 (4)	C14—C15—H15	119.4
C7—C6—H6	119.8	C15—C16—C17	121.5 (3)
C5—C6—H6	119.8	C15—C16—H16	119.3
C8—C7—C6	119.5 (3)	C17—C16—H16	119.3
C8—C7—H7	120.3	C16—C17—C18	118.3 (3)
C6—C7—H7	120.3	C16—C17—H17	120.9
C9—C8—C7	121.5 (4)	C18—C17—H17	120.9
C9—C8—H8	119.2	C17—C18—C19	120.8 (3)
C7—C8—H8	119.2	C17—C18—H18	119.6
C8—C9—C10	121.3 (4)	C19—C18—H18	119.6
C8—C9—H9	119.4	C18—C19—C14	120.9 (3)
C10—C9—H9	119.4	C18—C19—H19	119.5
C9—C10—C5	117.2 (3)	C14—C19—H19	119.5
C9—C10—C1	123.9 (3)		

### Hydrogen-bond geometry (Å, °)

**Table:**

*D*—H···*A*	*D*—H	H···*A*	*D*···*A*	*D*—H···*A*
N1—H1···O1	0.91 (1)	1.84 (3)	2.582 (3)	137 (3)
N2—H2···O1^i^	0.86	2.43	3.043 (3)	129

Symmetry codes: (i) *x*, *y*−1, *z*.
